# Global research trends and hotspots in blood-based biomarkers for Alzheimer’s disease: a bibliometric analysis

**DOI:** 10.3389/fnagi.2026.1886781

**Published:** 2026-09-29

**Authors:** Shengjie Wang, Junyao Zhang, Shaojun Sun, Renlong Xiao, Zhiyi Zhang, Junyan Yao

**Affiliations:** Department of Anesthesiology, Shanghai East Hospital, Tongji University School of Medicine, Shanghai, China

**Keywords:** Alzheimer’s disease, amyloid-beta, bibliometric analysis, blood-based biomarkers, phosphorylated tau, plasma

## Abstract

**Background:**

Blood-based biomarkers (BBMs) are rapidly reshaping the diagnostic and translational landscape of Alzheimer’s disease (AD), offering a scalable alternative to cerebrospinal fluid (CSF) assays and positron emission tomography (PET). This paper aims to conduct bibliometric analysis on the hotspots and trends in this field.

**Methods:**

Publications addressing AD-related blood/plasma/serum biomarkers from 1 January 2011 to 29 November 2025 were retrieved from the Web of Science Core Collection (WoSCC) and Scopus. Only English-language articles and reviews were included. VOSviewer, CiteSpace, and the R package bibliometrix were used to evaluate publication trends, collaboration networks (countries, institutions, authors), co-cited journals and references, and keyword evolution.

**Results:**

A total of 1,771 publications with 53,568 citations were included. Annual output rose steadily and accelerated after 2018, while 2025 records covered approximately the first 11 months of the year. The USA (795 publications) and China (478) were the most productive countries, whereas the UK and Sweden showed strong citation impact and collaboration intensity. The University of Gothenburg (263) and Sahlgrenska University Hospital (237) were the leading institutions. Henrik Zetterberg (218) and Kaj Blennow (185) were the most prolific authors. Co-citation analysis identified the NIA–AA biological research framework and validation studies of plasma amyloid-beta (Aβ) and plasma phosphorylated tau (p-tau) as the intellectual base. Keyword and burst analyses indicated a clear shift from early themes (e.g., proteomics and oxidative stress) toward clinically oriented topics, including plasma p-tau217/p-tau181, diagnostic performance, and implementation recommendations.

**Conclusion:**

Research on AD BBMs has entered a phase of rapid growth and clinical translation. Plasma phosphorylated tau (especially p-tau217) is the leading biomarker for detecting AD-specific pathology. Plasma Aβ ratios play a supportive role in amyloid triage, while neurodegeneration (NfL) and astroglial (GFAP) markers provide complementary information for prognosis and monitoring. Future priorities include real-world implementation, assay standardization, and integration of multi-marker panels into biological diagnostic/staging frameworks.

## 1 Introduction

Alzheimer’s disease (AD) is the most prevalent neurodegenerative cause of dementia and is characterized neuropathologically by extracellular Aβ plaques and intracellular neurofibrillary tangles composed of hyperphosphorylated tau. Historically, the clinical diagnosis of AD relied on symptom-based criteria (e.g., the NINCDS–ADRDA criteria) (McKhann et al., 1984), later refined by the National Institute on Aging–Alzheimer’s Association (NIA–AA) workgroups to incorporate biomarkers in diagnostic recommendations for AD dementia, mild cognitive impairment (MCI), and preclinical disease (McKhann et al., 2011; Albert et al., 2011; Sperling et al., 2011).

Biomarker-supported diagnosis has traditionally depended on cerebrospinal fluid (CSF) assays and PET imaging. CSF biomarkers and PET provide strong biological specificity but are limited by invasiveness (CSF), cost and infrastructure requirements (PET), and restricted accessibility in routine practice. Amyloid PET, including Pittsburgh Compound-B (PiB) and subsequent tracers, has been foundational for *in vivo* amyloid detection and validation of fluid biomarkers (Klunk et al., 2004; Johnson et al., 2013).

Over the past decade, blood-based biomarkers (BBMs) have moved from exploratory research to near-clinical deployment, driven by ultrasensitive analytical platforms such as single-molecule array (Simoa) technology (Rissin et al., 2010). Compared with CSF and PET, blood testing is minimally invasive, scalable for population screening, and suitable for repeated sampling for longitudinal monitoring. A systematic review and meta-analysis has highlighted the growing evidence base for blood and CSF biomarkers across the AD continuum (Olsson et al., 2016; Dark et al., 2024a; Dhauria et al., 2024; Liu et al., 2024).

Among candidate BBMs, two classes have become central to AD pathobiology: (i) plasma Aβ measures biomarkers (e.g., Aβ42/40 ratios) validated against amyloid PET (Nakamura et al., 2018; Schindler et al., 2019), and (ii) plasma phosphorylated tau (p-tau) species, now classified as T1 biomarkers for AD diagnosis under the revised NIA-AA criteria (Jack et al., 2024), and provide high discrimination between AD and non-AD neurodegenerative disorders (Janelidze et al., 2020; Thijssen et al., 2020; Karikari et al., 2020; Palmqvist et al., 2020; Ashton et al., 2021; Ashton et al., 2024; Thijssen et al., 2021). In addition, biomarkers of neurodegeneration and astroglial activation—such as neurofilament light chain (NfL) and glial fibrillary acidic protein (GFAP)—provide complementary information for staging and prognosis (Janelidze et al., 2020; Mattsson et al., 2017; Benedet et al., 2021).

These biomarker advances have paralleled conceptual developments in how AD is defined. The NIA–AA Research Framework proposed a biological definition of AD based on the A/T/N classification system (Jack et al., 2018). More recently, revised criteria for diagnosis and staging have further emphasized biological and staging constructs aligned with biomarker evidence and potential clinical implementation pathways (Jack et al., 2024). In the treatment arena, the emergence of disease-modifying therapies (DMTs) targeting amyloid, such as lecanemab and donanemab, has increased demand for accessible biomarker-based triage and monitoring tools (van Dyck et al., 2023; Mintun et al., 2021).

Bibliometric analysis provides quantitative, reproducible approaches to map a scientific field by examining publication outputs, collaboration patterns, co-citation structures, and keyword dynamics. By integrating mapping tools (VOSviewer, CiteSpace) and R-based science mapping (bibliometrix), bibliometric studies can identify influential contributors and detect emerging frontiers. Therefore, we conducted a comprehensive bibliometric analysis of AD BBM research (1 January 2011 to 29 November 2025) to clarify global trends, core knowledge bases, and evolving hotspots, and to inform future research and clinical translation.

## 2 Materials and methods

### 2.1 Data source and search strategy

To ensure a comprehensive and robust bibliometric analysis, we searched two complementary databases: the Web of Science Core Collection (WoSCC) and Scopus. WoSCC provides curated, high-impact journal coverage with rigorous indexing standards, while Scopus offers broader interdisciplinary coverage. The search was performed on 29 November 2025 using the following strategy. For WoSCC, the search query combined terms for Alzheimer‘s disease (“Alzheimer’s disease” OR “Alzheimer disease”) with terms for blood-based biomarkers, including broad categories (“blood biomarker*,” “plasma biomarker*,” “serum biomarker*”) and specific analytes of interest (“phosphorylated tau,” “p-tau,” “plasma amyloid,” “Aβ,” “neurofilament light,” “NfL,” “GFAP,” “glial fibrillary acidic protein”). For Scopus, an equivalent query was applied using TITLE-ABS-KEY fields. The complete search syntax with full Boolean operators, field codes, and database-specific adaptations is provided in Supplementary Table S1. Inclusion criteria were: English language; published between 1 January 2011 and 29 November 2025; and studies related to Alzheimer’s disease and its blood biomarkers (plasma, serum, or whole blood). Letters, conference abstracts, proceedings, and non-article types were excluded. Bibliographic data (title, authors, affiliations, country, keywords, journal, citation counts, etc.) were exported as plain text in “full record and cited references” format. Two independent reviewers screened the records; disagreements were resolved by discussion or by consulting a third reviewer when necessary.

### 2.2 Screening and data preprocessing

The initial search yielded 1,851 records from WoSCC and 1,932 records from Scopus. From the WoSCC records, non-article/review records (*n* = 87), non-English records (*n* = 7), duplicates within WoSCC (*n* = 1), and irrelevant records after content screening (*n* = 35) were excluded, leaving 1,721 publications. From the Scopus records, non-article/review records (*n* = 108), non-English records (*n* = 30), duplicates within Scopus (*n* = 2), and irrelevant records (*n* = 54) were excluded, leaving 1,738 publications. The two sets were then merged, and inter-database duplicates were removed, resulting in a final total of 1,771 publications included for bibliometric analysis (Figure 1).

**Figure 1:**
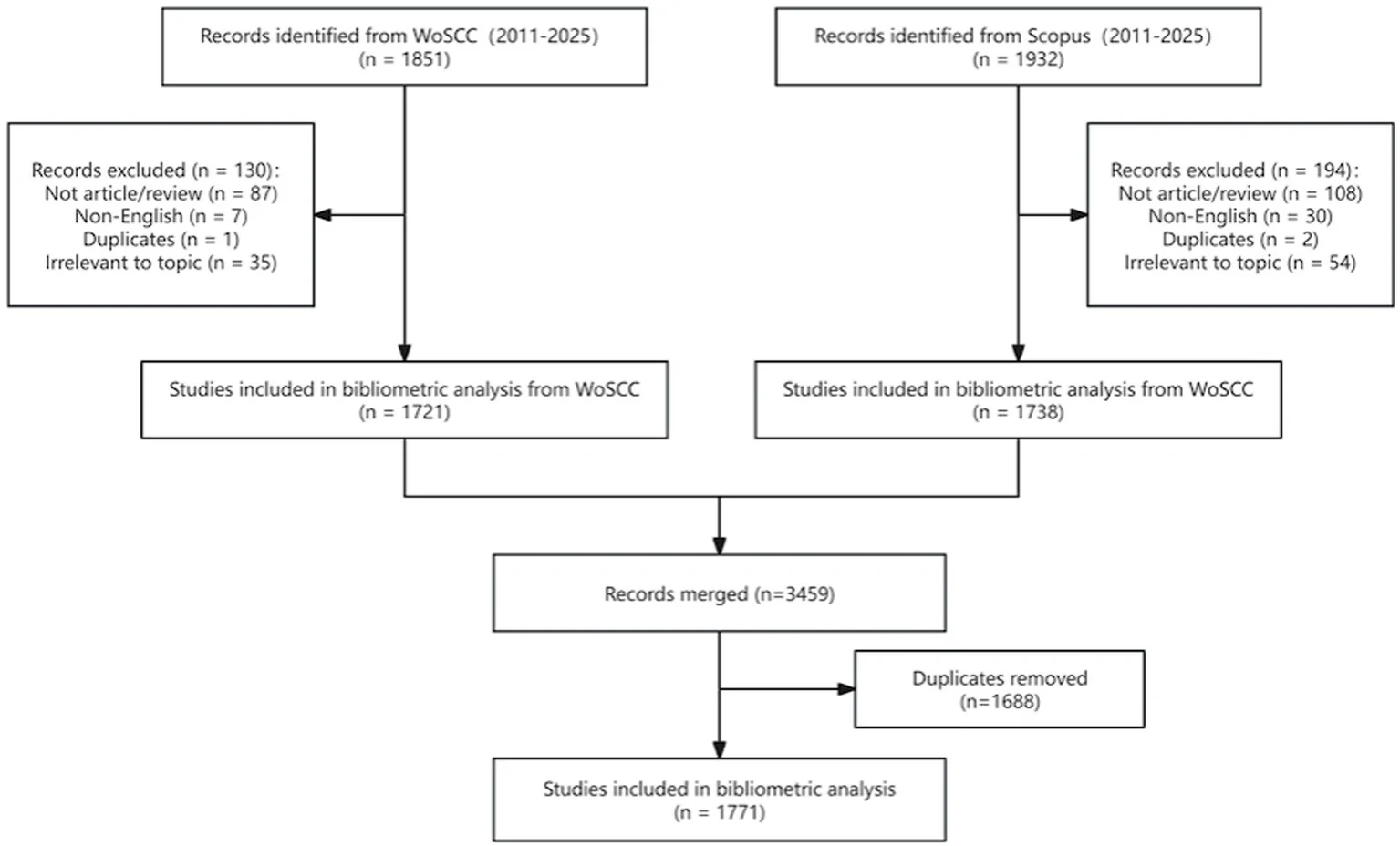
Flow diagram of literature retrieval and screening for the bibliometric analysis.

### 2.3 Bibliometric analysis and visualization tools

In the data analysis phase, we implemented a multi-platform workflow using Microsoft Excel 2019 for data management, VOSviewer (v1.6.20) for co-authorship and keyword networks, CiteSpace (v6.4R1) for co-citation clustering and burst detection, and R/bibliometrix (v4.5.2) for descriptive statistics and thematic evolution, supplemented by SCImago Graphica (v1.0.53) for geographic visualization mapping.

## 3 Results

### 3.1 Publication output and citation growth

After merging the records from WoSCC and Scopus and removing duplicates, a total of 1,771 publications related to AD BBMs were included for analysis, accumulating 53,568 citations. The annual number of publications increased steadily over the study period and accelerated markedly after 2018 (Figure 2). Notably, 2025 publications were retrieved up to late November, and therefore the full-year output for 2025 is likely underestimated.

**Figure 2:**
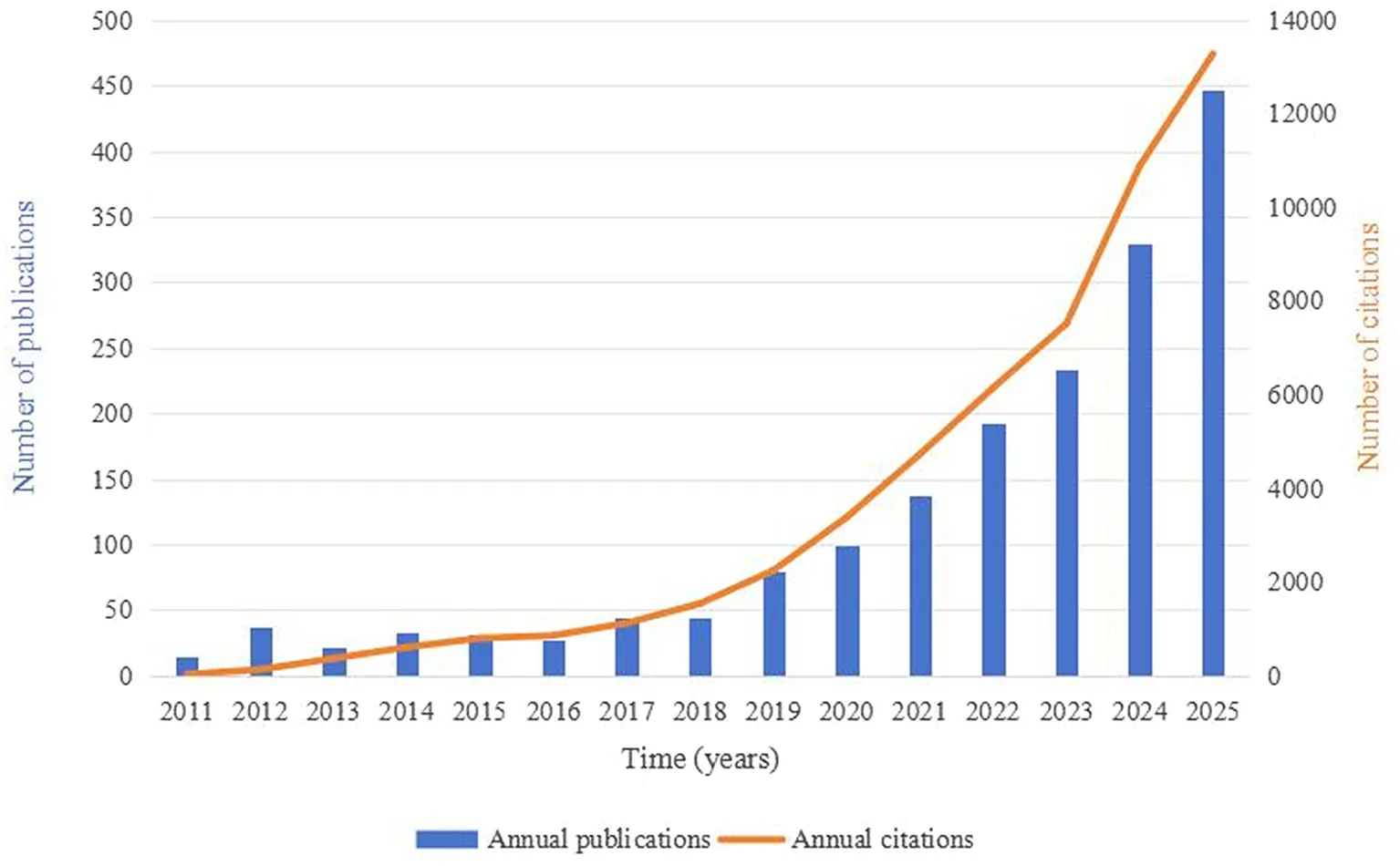
Annual publication output and citations of studies on AD BBMs (2011–2025). Bars represent the number of publications per year and the line represents total citations per year.

### 3.2 Country/region contributions and collaboration

Country collaboration mapping (minimum ≥5 publications) included 46 countries/regions, indicating broad global participation. The USA was the most productive country (795 publications), followed by China (478), the UK (453), and Sweden (376) (Table 1). In terms of collaboration intensity (total link strength, TLS), the UK (TLS = 1,484) and the USA (TLS = 1,378) occupied central positions, highlighting their roles as major international collaboration hubs. VOSviewer and CiteSpace maps (Figures 3A–C) consistently showed that the USA, UK, and Sweden formed the central collaboration backbone, while China emerged as the most productive Asian country with gradually strengthening but still weaker cross-regional links. Temporal visualization (Figure 3D) revealed a clear expansion of collaboration networks from a transatlantic core to Asia, Latin America, and the Middle East after 2016.

**Table 1:** Top 10 productive countries/regions in AD BBM research (2011–2025).

Country	Documents	Citations	Total Link strength
The US	795	28,277	1,378
China	478	9,298	908
The UK	453	23,817	1,484
Sweden	376	20,614	1,332
France	162	6,155	666
Spain	159	6,936	431
Germany	126	5,816	423
Italy	126	5,507	305
Australia	86	5,062	337
Canada	86	5,062	352

**Figure 3:**
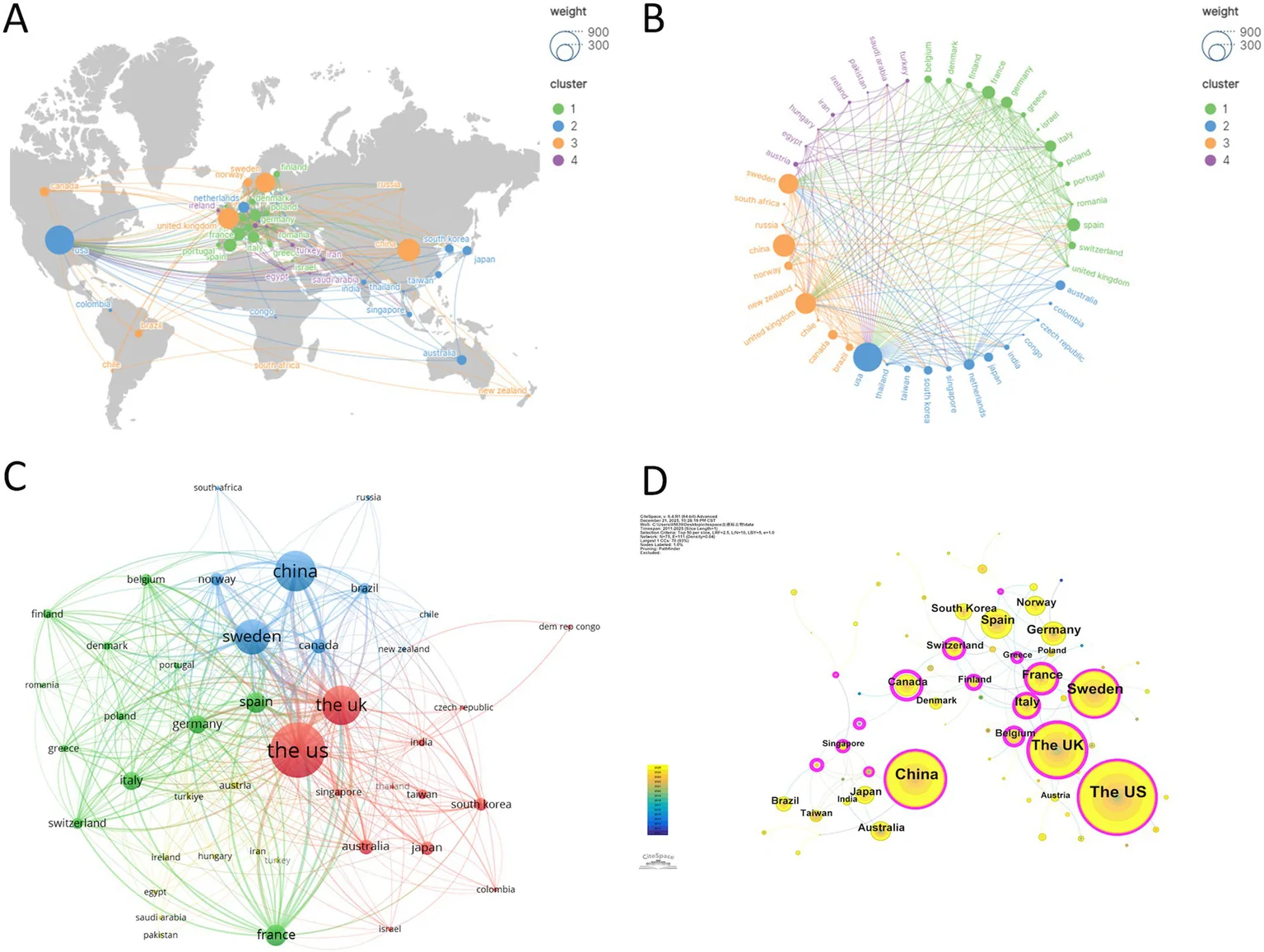
Country/regional collaboration networks and temporal evolution in AD BBM research (2011–2025). **(A)** Global collaboration map of countries/regions in AD BBM research generated with VOSviewer and Scimago Graphica. Node size reflects publication volume, link thickness indicates collaboration strength, and colors represent collaboration clusters. **(B)** Country/region co-authorship network visualization (VOSviewer and Scimago Graphica). Countries/regions with ≥5 publications were included. Total link strength (TLS) quantifies collaboration intensity. **(C)** Country/region collaboration network generated using CiteSpace (2011–2025, 1-year slice). Node rings indicate time-sliced contributions; purple trims indicate high betweenness centrality; links represent collaborations. **(D)** Temporal evolution of country/region collaboration (CiteSpace). Colors represent publication time slices from 2011 to 2025, illustrating the expansion of international collaborations over time.

### 3.3 Institutional productivity and networks

Institutional analysis (minimum ≥15 publications) included 85 institutions. The University of Gothenburg (263 publications) and Sahlgrenska University Hospital (237) were the leading contributors, followed by University College London (UCL; 201) and the Hong Kong Center for Neurodegenerative Diseases (150) (Table 2). VOSviewer and CiteSpace maps demonstrated dense collaborations among leading European and North American centers, with several institutions acting as cross-network bridges (Figures 4A–C).

**Table 2:** Top 10 productive institutions in AD BBM research (2011–2025).

Organization	Documents	Citations	Total link strength
University of Gothenburg	263	15,016	1923
Sahlgrenska University Hospital	237	11,976	1846
University College London	201	8,635	1,484
Hong Kong Centre for Neurodegenerative Diseases	150	4,753	1,290
UCL Institute of Neurology	139	12,255	967
King’s College London	130	7,628	1,025
Lund University	91	6,954	599
University of Pittsburgh	78	4,011	541
University of Wisconsin	73	1,405	599
Skane University Hospital	70	5,712	486

**Figure 4:**
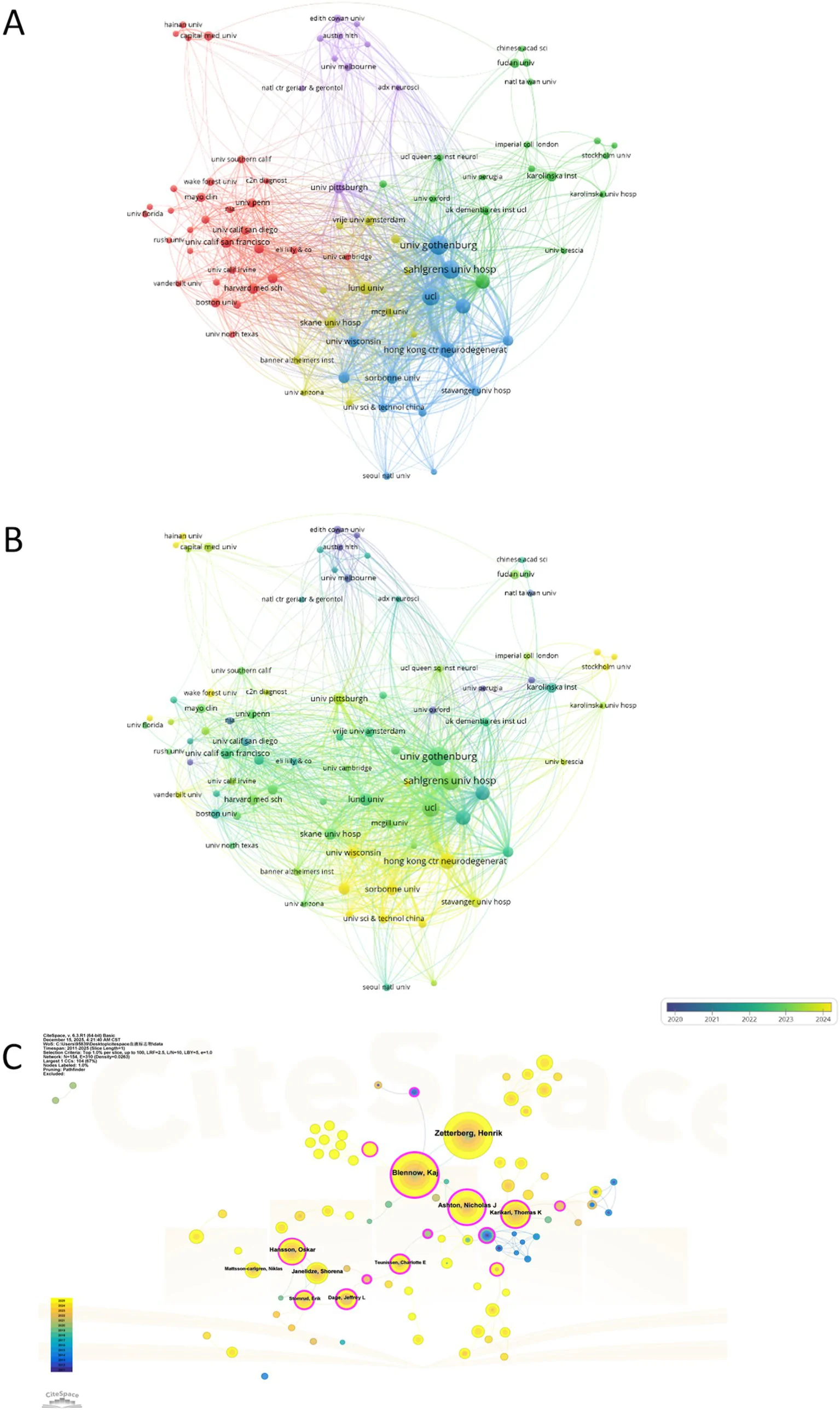
Institutional collaboration networks and temporal evolution in AD BBM research (2011–2025). **(A)** Institutional collaboration network (VOSviewer) for institutions with ≥15 publications. Node size indicates number of publications; link thickness indicates collaboration strength; colors represent clusters. **(B)** Overlay visualization of institutional collaborations (VOSviewer). Colors indicate the average publication year (recent activity shown toward the high end of the scale), highlighting emerging institutions. **(C)** Institutional collaboration network generated with CiteSpace (2011–2025). Nodes represent institutions; ring colors indicate time slices; purple rims denote higher centrality.

### 3.4 Author productivity and collaboration

Author co-authorship mapping (minimum ≥12 publications) included 80 authors. Henrik Zetterberg (218 publications) and Kaj Blennow (185) were the most prolific and highly connected authors (Table 3), forming a central hub linked to multiple international teams. Other high-output authors included Nicholas J. Ashton, Oskar Hansson, and Thomas K. Karikari. Network visualizations (Figures 5A,B) illustrate several collaborative clusters, reflecting cohort-based and platform-based research communities.

**Table 3:** Top 10 productive authors in AD BBM research (2011–2025).

Author	Documents	Citations	Total link strength
Henrik Zetterberg	218	13,146	722
Kaj Blennow	185	12,188	704
Nicholas J. Ashton	93	5,154	470
Oskar Hansson	70	6,502	365
Thomas K. Karikari	53	4,032	257
Shorena Janelidze	52	4,525	289
Jeffrey L. Dage	35	2,840	120
Erik Stomrud	34	3,518	234
Charlotte E. Teunissen	34	1,597	91
Niklas Mattsson - Carlgren	32	3,183	233

**Figure 5:**
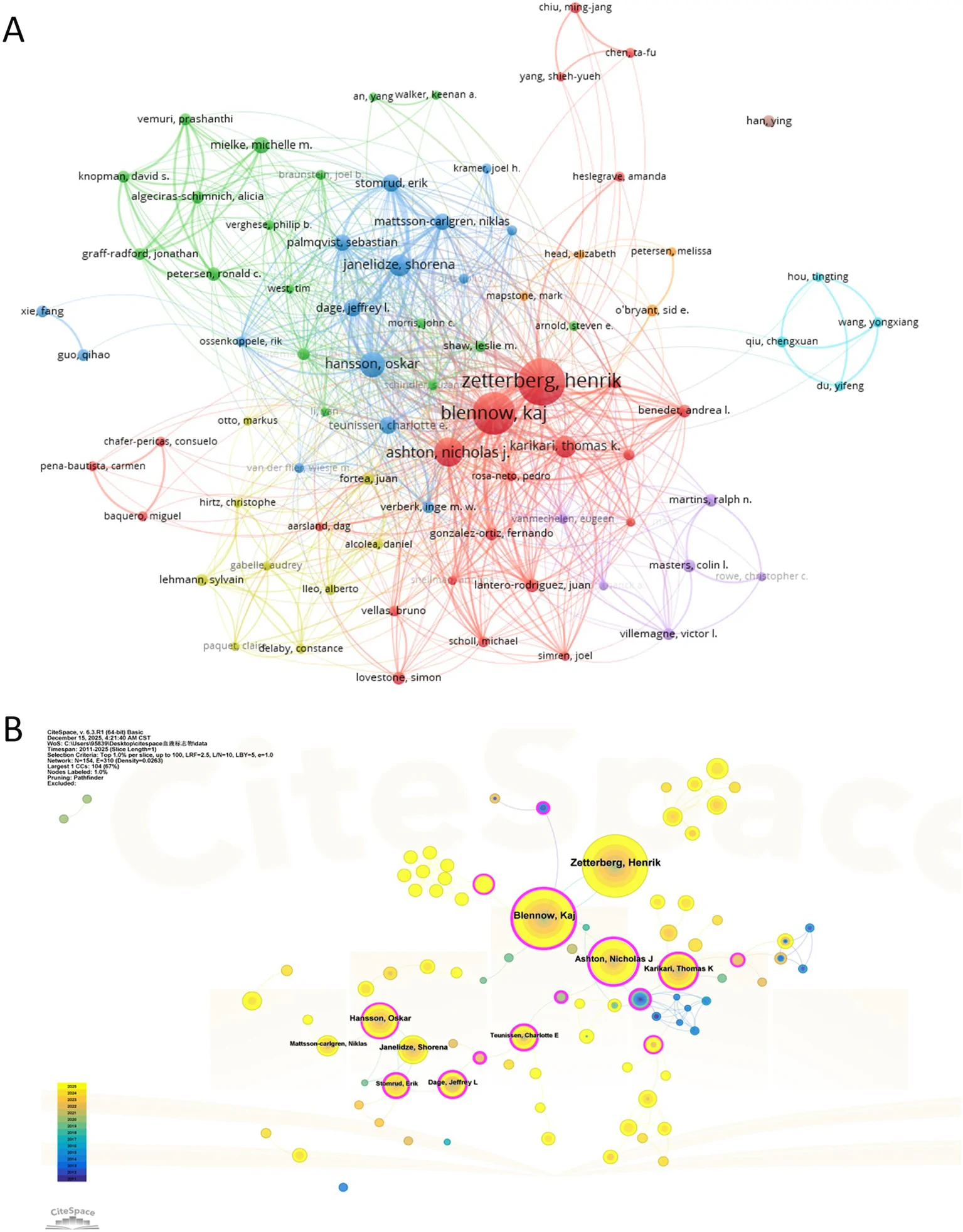
Author collaboration networks in AD BBM research (2011–2025). **(A)**Author co-authorship network (VOSviewer) for authors with ≥12 publications. Node size indicates publication count; link thickness reflects collaboration strength; colors denote co-authorship clusters. **(B)** Author collaboration network (CiteSpace, 2011–2025). Node rings represent temporal contributions; highly central authors are highlighted by purple rims.

### 3.5 Journal landscape and co-cited sources

Co-cited journal analysis identified the major disciplinary outlets shaping AD BBM research. The most frequently co-cited journals were Alzheimer’s & Dementia (8,483 co-citations), Neurology (4,820), Journal of Alzheimer’s Disease (4,151), JAMA Neurology (2,901), and Alzheimer’s Research & Therapy (2,835) (Table 4). Network mapping indicated several journal clusters spanning neurology, neuroimaging, biomarker methodology, and general medicine (Figures 6A,B). The dual-map overlay further suggested that publications in molecular/biology and clinical medicine journals frequently cite foundational work in neurology, immunology, and general medicine, reflecting interdisciplinary knowledge flow (Figure 6C).

**Table 4:** Top 10 co-cited journals (sources) in AD BBM research (2011–2025).

Source	Citations	Total Link strength
Alzheimers and Dementia	8,483	550,076
Neurology	4,820	372,705
Journal of Alzheimer’s Disease	4,151	327,020
JAMA Neurology	2,901	212,093
Alzheimer’s Research and Therapy	2,835	211,290
The Lancet Neurology	2,567	191,587
Brain	2,428	191,679
Nature Medicine	2,144	147,255
Neurobiology of Aging	1822	156,472
Annals of Neurology	1,448	137,012

**Figure 6:**
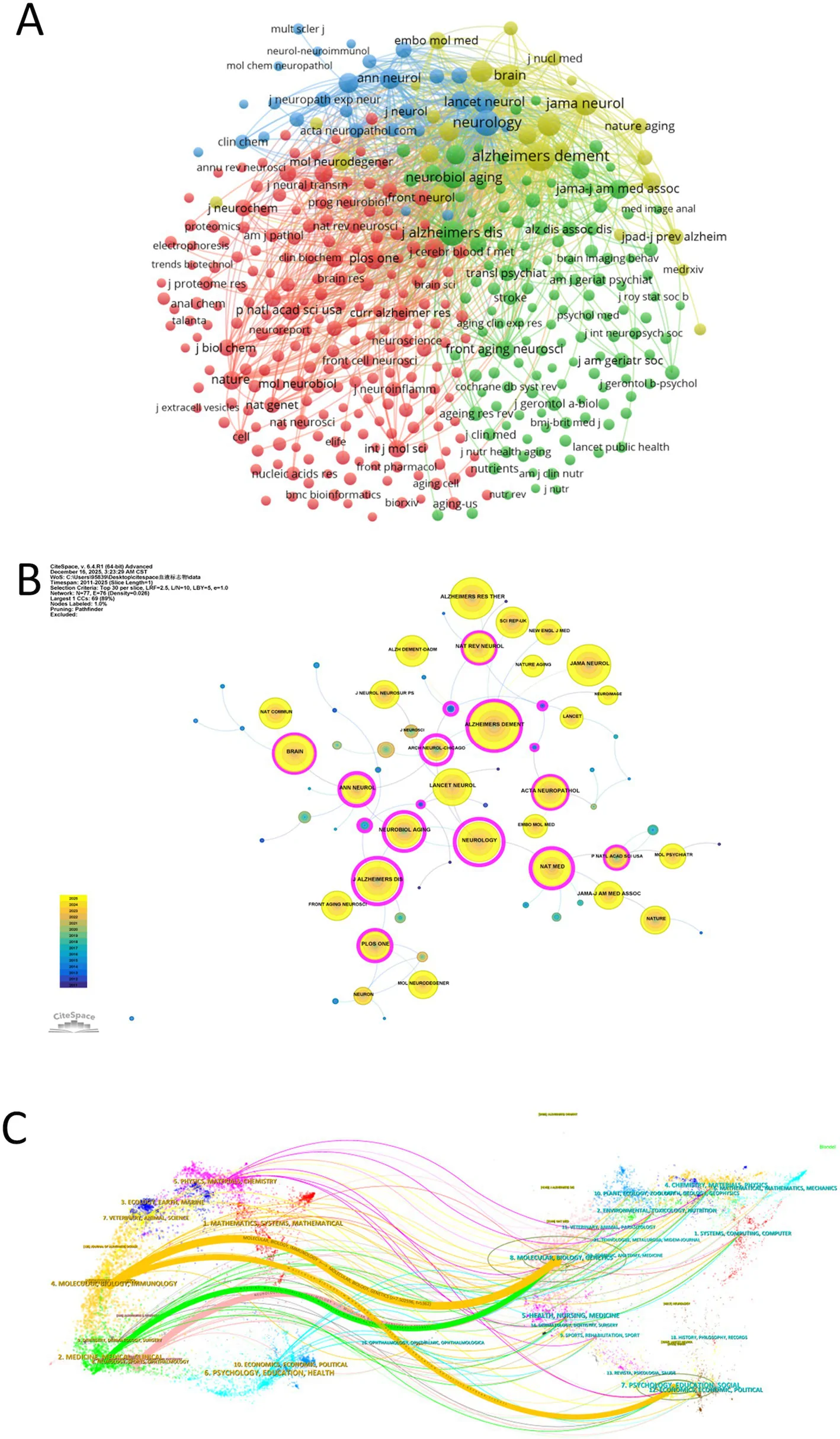
Co-cited journal networks and interdisciplinary knowledge flow in AD BBM research (2011–2025). **(A)** Co-cited journal network (VOSviewer) based on cited-source analysis (minimum ≥30 citations). Node size reflects co-citation frequency and link thickness indicates relatedness. **(B)** Co-cited journal network generated using CiteSpace (2011–2025). Rings denote temporal co-citation patterns; purple rims indicate higher centrality. **(C)** Dual-map overlay of journals (CiteSpace). Left side represents citing journals and right side represents cited journals; colored paths indicate major citation trajectories across disciplines.

### 3.6 Co-cited references and knowledge base

Co-cited reference analysis (minimum ≥20 citations) included 484 key references and revealed the intellectual structure of the field. The most influential references were the NIA–AA Research Framework (Jack et al., 2018) and biomarker validation studies of plasma p-tau and plasma Aβ (Table 5). VOSviewer and CiteSpace clustering identified multiple thematic clusters, such as plasma p-tau217, neurofilament light, diagnostic performance/accuracy, proteomics markers, and imaging-related studies (Figures 7A–C). These clusters collectively indicate that the field has transitioned from exploratory biomarker discovery to clinically oriented validation and implementation.

**Table 5:** Top 10 co-cited references in AD BBM research (2011–2025).

Cited reference	Citations	Total Link strength
Jack CR jr, 2018, Alzheimers Dement, v14, p. 535, doi 10.1016/j.jalz.2018.02.018	484	10,782
Mckhann GM, 2011, Alzheimers Dement, v7, p263, doi 10.1016/j.jalz.2011.03.005	386	6,623
Karikari TK, 2020, Lancet Neurol, v19, p422, doi 10.1016/s1474-4422(20)30071-5	331	8,380
Janelidze S, 2020, Nat Med, v26, p379, doi 10.1038/s41591-020-0755-1	312	8,227
Nakamura A, 2018, Nature, v554, p249, doi 10.1038/nature25456	307	7,503
Palmqvist S, 2020, Jama JAM Med Assoc., v324, p772, doi 10.1001/jama.2020.12134	292	8,259
Schindler SE, 2019, Neurology, v93, pe1647, doi 10.1212/wnl.0000000000008081	233	6,482
Olsson B, 2016, Lancet Neurol, v15, p673, doi [10.1016/s1474-4422(16)00070-3 10.1016/s1474-4422(16)00070-3]	223	5,215
Albert MS, 2011, Alzheimers Dement, v7, p270, doi 10.1016/j.jalz.2011.03.008	220	4,155
Mckhann g, 1984, Neurology, v34, p939, doi 10.1212/wnl.34.7.939	198	2,974

**Figure 7:**
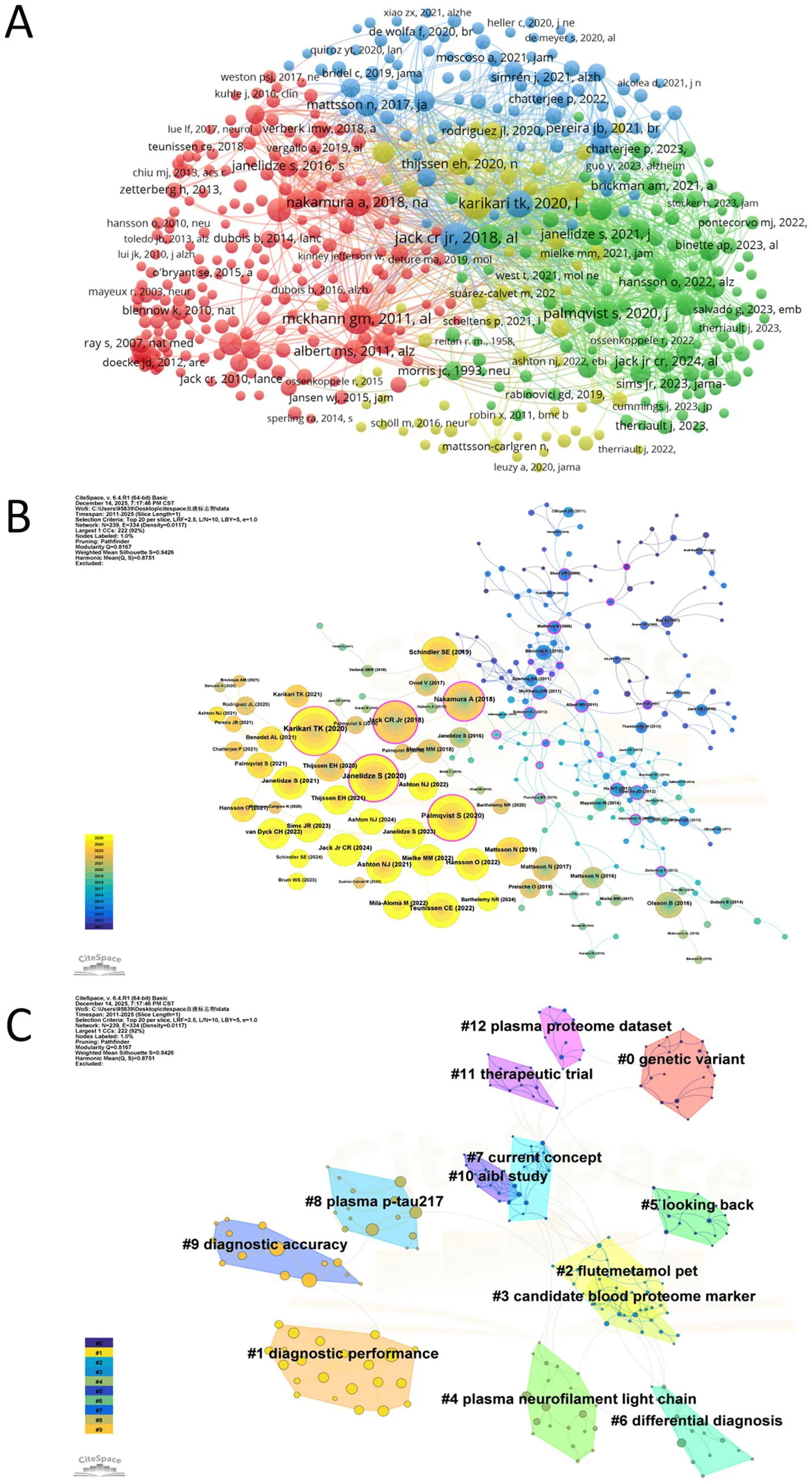
Co-cited reference networks and thematic clusters in AD BBM research (2011–2025). **(A)** Co-cited reference network (VOSviewer) based on cited-reference analysis (minimum ≥20 citations). Nodes represent references; size indicates co-citation frequency; colors represent clusters of related literature. **(B)** Co-cited reference network (CiteSpace, 2011–2025). Ring colors denote time slices; purple rims indicate higher betweenness centrality; the network highlights influential landmark studies. **(C)** Major co-citation clusters of references identified by CiteSpace. Cluster labels (e.g., plasma p-tau217, neurofilament light chain, diagnostic performance, proteome markers, genetic variants) indicate thematic concentrations.

### 3.7 Keyword co-occurrence, burst detection, and topic evolution

Keyword analysis (minimum ≥10 occurrences) extracted 99 high-frequency terms and revealed major hotspots. Co-occurrence networks emphasized plasma biomarkers Aβ tau/p-tau, MCI neurodegeneration, and assay technologies (e.g., Simoa) (Figures 8A,B). CiteSpace keyword clustering and timeline mapping demonstrated thematic evolution from early work emphasizing CSF biomarkers oxidative stress and proteomics toward plasma biomarkers diagnostic recommendations neurofilament light and clinical performance metrics (Figures 8C–E). Keyword burst analysis further highlighted changing research frontiers with recent bursts related to “blood”, “performance”, and “pathology” (Figure 8F). Bibliometrix visualizations showed how leading authors keywords nd journals are connected (Figure 8G) and illustrated emerging trend topics with p-tau217 and early detection rising in recent years (Figure 8H).

**Figure 8:**
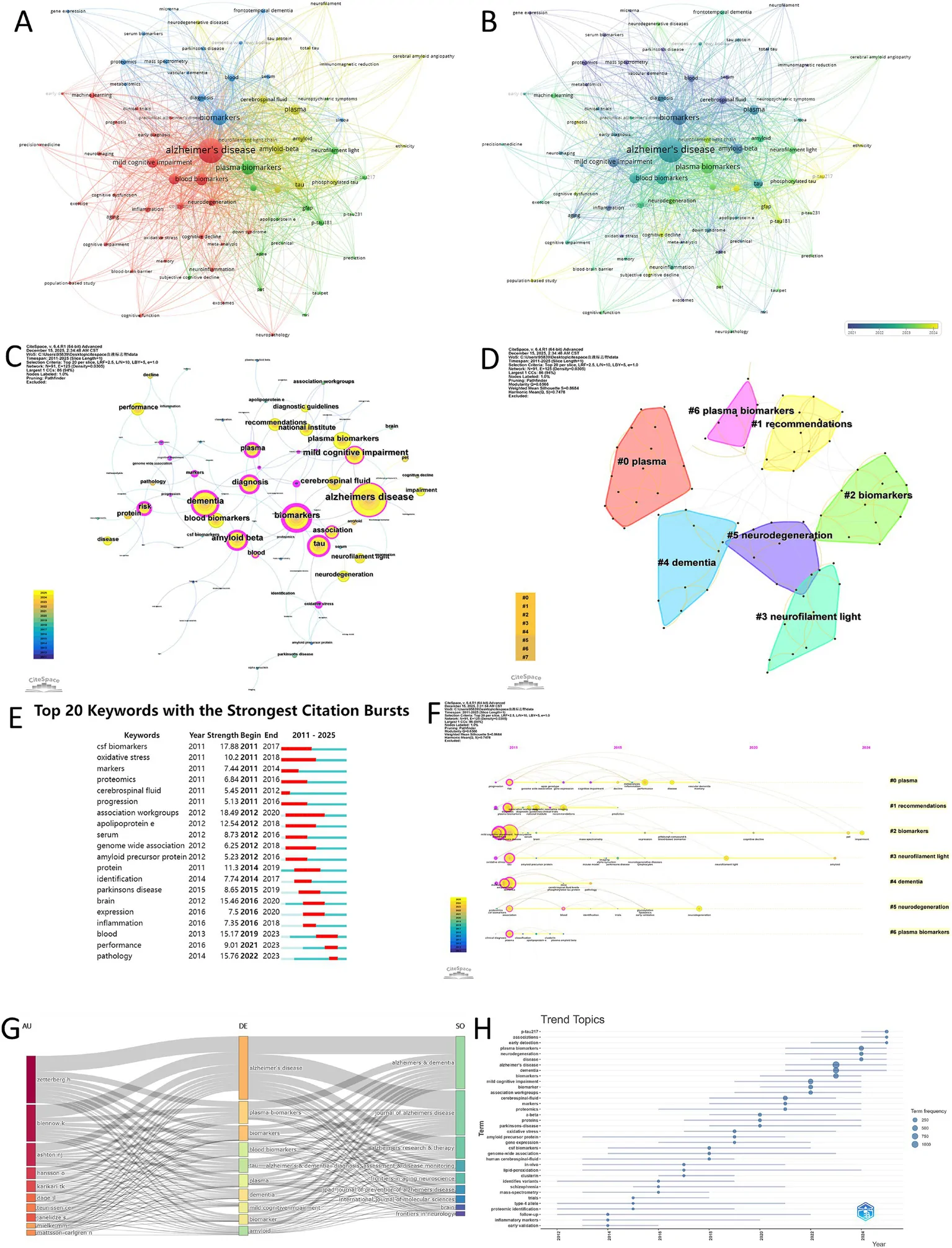
Keyword co-occurrence, thematic evolution, and research frontiers in AD BBM research (2011–2025). **(A)** Keyword co-occurrence network (VOSviewer) for keywords with ≥10 occurrences. Node size indicates frequency; links indicate co-occurrence; colors represent thematic clusters (e.g., tau/p-tau, amyloid, proteomics/machine learning). **(B)** Overlay visualization of keyword co-occurrence (VOSviewer). Colors indicate the average publication year, highlighting newer hotspots and clinical implementation terms. **(C)** Keyword co-occurrence network generated using CiteSpace (2011–2025). Node size reflects frequency; ring colors represent time slices; purple rims indicate higher centrality keywords bridging topics. **(D)** Keyword clusters identified by CiteSpace. Clusters such as “plasma,” “recommendations,” “neurofilament light,” and “neurodegeneration” summarize major thematic communities. **(E)** Top 20 keywords with the strongest citation bursts (CiteSpace). Red bars indicate burst periods; burst strength reflects the intensity of increased attention over time. **(F)** Timeline view of keyword clusters (CiteSpace). Keywords are arranged by cluster on the right; the horizontal axis represents time, illustrating topic emergence and persistence. **(G)** Three-field plot (bibliometrix) linking top authors (AU), keywords (DE),and sources (SO). Ribbon width indicates the strength of associations among authors, themes, and journals. **(H)** Trend topics (bibliometrix) across years. Bubble size represents term frequency and horizontal lines indicate active periods, highlighting recent emphasis on p-tau217 and early detection.

## 4 Discussion

### 4.1 Rapid growth and research distribution of AD BBMs

In this bibliometric analysis based on WoSCC and Scopus, we systematically mapped the intellectual base and evolving hotspots of BBMs for AD from 2011–2025, including 1,771 articles or reviews. Our results show that research on BBMs for AD has transitioned from an exploratory phase to a translational phase with a clear acceleration after 2018. This inflection is biologically and methodologically plausible. Conceptually, the NIA–AA Research Framework formalized a biological definition of AD and provided the A/T/N scheme to align biomarkers with core pathophysiology (Jack et al., 2018). More recently, revised criteria for diagnosis and staging further encouraged a structured, biomarker-informed approach and clarified how biomarkers may be used to define disease stages beyond clinical syndromes (Jack et al., 2024). Methodologically, ultrasensitive platforms enabled reliable quantification of low-abundance analytes in plasma, removing a key technical barrier that had historically limited BBM feasibility (Rissin et al., 2010). In clinical practice, the emergence of amyloid targeted disease-modifying therapies, including lecanemab and donanemab, shifting treatment strategies from traditional symptom improvement to targeted etiology, increases the demand for AD BBMs to identify patients with potential amyloid pathology and monitor treatment-related changes and safety (van Dyck et al., 2023; Mintun et al., 2021).

From a specific perspective, Country and collaboration mapping indicated that the United States and China contributed the largest publication volumes, whereas several European countries achieved high citation impact relative to output. Country and institution networks demonstrated that the USA, the UK, and Sweden form a densely connected collaboration backbone, with high publication output and link strength. The prominence of the University of Gothenburg and Sahlgrenska University Hospital is consistent with the long-standing expertise of these centers in neurochemistry and longitudinal cohort studies, and with the role of Scandinavian cohorts in validating plasma p-tau biomarkers. The increasing contributions from China and other regions highlight global expansion, but network maps suggest that strengthening cross-regional collaboration and harmonization initiatives could further improve generalizability across diverse populations.

### 4.2 Research trend of AD BBMs

Co-citation analysis suggests that the intellectual base of AD BBM research rests on two mutually reinforcing pillars. The first comprises biomarker-informed diagnostic and staging frameworks that conceptualize AD as a continuum defined by amyloid, tau, and neurodegeneration processes (AT(N)), thereby defining what blood biomarkers should approximate (Jack et al., 2018, 2024). The second includes landmark cross-modality validation studies benchmarking plasma assays against PET/CSF and evaluating diagnostic discrimination across AD and non-AD neurodegenerative disorders (Nakamura et al., 2018; Schindler et al., 2019; Janelidze et al., 2020; Thijssen et al., 2020; Karikari et al., 2020; Palmqvist et al., 2020; Ashton et al., 2021; Ashton et al., 2024; Teunissen et al., 2022; Hansson et al., 2023).

Burst detection and timeline analyses provide a nuanced view of how the field has evolved. Early bursts (e.g., oxidative stress, proteomics, CSF biomarkers) reflect exploratory work and the search for peripheral signatures (Dong et al., 2024). Later bursts (e.g., blood, performance, pathology) and clusters labeled “recommendations” and “diagnostic performance” reflect the current translational phase, in which studies emphasize real-world accuracy, cutoffs, and implementable workflows. This temporal shift is consistent with the maturation pathway described in implementation-focused reviews (Teunissen et al., 2022; Hansson et al., 2023).

Our hotspot analyses show a shift from exploratory peripheral signatures (e.g., inflammatory pathways and untargeted proteomics) to a focused set of pathophysiology-linked plasma biomarkers with stronger reproducibility. Phosphorylated tau species (p-tau217, p-tau181, p-tau231) emerged as prominent recent hotspots, consistent with evidence that they track AD-related tau pathology and can differentiate AD from other neurodegenerative disorders with high accuracy in well-characterized cohorts (Ashton et al., 2021; Ashton et al., 2024; Hansson et al., 2023). Plasma Aβ42/Aβ40 (and related ratios) remains central, particularly for amyloid pathology triage, but continues to face assay harmonization challenges and context-dependent performance across platforms and laboratories (Nakamura et al., 2018; Schindler et al., 2019; Teunissen et al., 2022; Sarto et al., 2024). Finally, markers reflecting astroglial activation (GFAP) and axonal injury (NfL) appear to be increasingly used as complements that support prognostic stratification and longitudinal monitoring rather than stand-alone etiologic diagnosis (Mattsson et al., 2017; Benedet et al., 2021; Teunissen et al., 2022; Hansson et al., 2023).

### 4.3 Plasma phosphorylated tau as the dominant BBM signal

A central finding of our hotspot mapping is the dominance of plasma p-tau, consistent with a growing body of evidence that p-tau species in plasma can discriminate AD from non-AD neurodegenerative disorders with high accuracy when validated against PET/CSF benchmarks. Multiple large studies and multi-cohort evaluations established plasma p-tau181 as a sensitive and relatively specific marker of AD pathophysiology and progression (Janelidze et al., 2020; Thijssen et al., 2020; Karikari et al., 2020). For example, Janelidze et al. demonstrated that plasma p-tau181 relates to other biomarkers, supports differential diagnosis, and tracks longitudinal progression toward AD dementia (Janelidze et al., 2020). Thijssen et al. further highlighted diagnostic value in distinguishing AD from frontotemporal lobar degeneration, reinforcing clinical relevance in memory clinics where phenotypic overlap is common (Thijssen et al., 2020). These studies help explain why p-tau181 became a foundational node in both co-citation and keyword networks.

Yet, the field is not static: bibliometric bursts increasingly favor p-tau217, reflecting consistent observations that p-tau217 may outperform p-tau181 in detecting AD pathology and separating AD from other neurodegenerative diseases. Palmqvist et al. reported strong discriminative accuracy for plasma p-tau217 across neurodegenerative disorders, and subsequent multicenter work supported its robustness and clinical promise (Palmqvist et al., 2020; Thijssen et al., 2021; Cecchetti et al., 2024; Feizpour et al., 2024; Khalafi et al., 2025; Mendes et al., 2024; Palmqvist et al., 2024; Therriault et al., 2024). More recently, cohort studies have evaluated commercially available p-tau217 immunoassays with accuracy comparable to CSF biomarkers for identifying abnormal Aβ and tau pathologies, which is a major step toward scalable deployment beyond expert research laboratories (Ashton et al., 2024). The prominence of these works in our timeline view suggests a “second phase” of BBM research: moving from proof-of-concept assays to platforms that can be reproduced, distributed, and quality-controlled. A further refinement is the emergence of p-tau231 as a candidate marker with potential sensitivity to early AD pathophysiology. Ashton et al. proposed that plasma p-tau231 may rise early and provide complementary information across the disease continuum (Ashton et al., 2021).

Nevertheless, several uncertainties remain. First, plasma p-tau dynamics across the disease spectrum are complex and depend on reference standards, cohorts, and analytical platforms. Longitudinal modeling suggests that plasma p-tau trajectories relate to amyloid accumulation and predict downstream tau pathology, but the timing relative to other biomarkers can vary by methodology and cohort characteristics (Moscoso et al., 2021). Second, although p-tau species show impressive group-level performance, individual-level interpretation requires carefully validated cutoffs and uncertainty management, particularly in the “intermediate zone” of borderline results. Third, p-tau is not a standalone solution: AD commonly coexists with vascular and other pathologies, and the relationship between clinical symptoms and AD biomarker positivity is modulated by resilience, cognitive reserve, and comorbidities—factors explicitly acknowledged in modern staging perspectives (Jack et al., 2024). For these reasons, our bibliometric synthesis supports a view of plasma p-tau as the anchor of BBM-based diagnosis, but not the entire diagnostic architecture.

### 4.4 Plasma amyloid biomarkers and integrated triage algorithms for scalable diagnosis and treatment pathways

Although p-tau dominates recent hotspots, our co-citation base and cluster structure indicate that plasma amyloid measurement remains a foundational theme. High-precision assays for plasma Aβ42/40—especially approaches leveraging immunoprecipitation and mass spectrometry—showed that blood can track brain amyloidosis with clinically meaningful accuracy, helping challenge the long-standing assumption that peripheral Aβ noise would preclude diagnostic utility (Nakamura et al., 2018; Schindler et al., 2019). For instance, Nakamura et al. reported high-performance plasma amyloid biomarkers for detecting brain amyloid pathology (Nakamura et al., 2018), and Schindler et al. demonstrated that plasma Aβ42/40 can predict current and future brain amyloidosis (Schindler et al., 2019; Assfaw et al., 2024; Vogelgsang et al., 2024). These works are prominent in our co-citation network, reflecting their role as methodological breakthroughs.

A practical implication is that plasma Aβ may be most useful in a stepwise strategy: as a first-pass indicator in certain populations or as part of a combined panel where p-tau provides disease specificity and Aβ supports biological staging (Jack et al., 2018, 2024; Teunissen et al., 2022). The clinical logic has strengthened with the development of anti-amyloid therapies for early AD, which require evidence of amyloid pathology and benefit from efficient referral workflows (van Dyck et al., 2023; Mintun et al., 2021). From a health-system perspective, plasma tests can function as capacity multipliers—reducing the number of costly PET scans and invasive lumbar punctures while shortening time-to-diagnosis.

### 4.5 Neurodegeneration and astroglial markers: complementary roles for prognosis and disease monitoring

Our keyword and cluster analyses also highlight neurodegeneration- and glia-related terms, consistent with an expanded view of BBMs as tools not only for etiologic classification but also for staging and monitoring. Plasma neurofilament light (NfL) is a sensitive marker of neuroaxonal injury that correlates with neurodegeneration but is not specific to AD (Mattsson et al., 2017; Dark et al., 2024b; Gotze et al., 2024; Jung and Damoiseaux, 2024; Lehmann et al., 2023). Its strength lies in capturing intensity and dynamics of neuronal damage—useful for prognosis, differential diagnosis in broader neurology contexts, and potentially for monitoring downstream neurodegenerative impact.

Astroglial activation markers, particularly glial fibrillary acidic protein (GFAP), have gained attention as indicators that may rise early in the presence of amyloid pathology and reflect reactive gliosis (Benedet et al., 2021). Benedet et al. (2021) reported differences between plasma and CSF GFAP across the AD continuum and associations with disease-related biology, reinforcing the concept that glial markers may add information beyond Aβ and tau (Goschel et al., 2024; Holper et al., 2024; Zou et al., 2024). The bibliometric emergence of GFAP-related hotspots likely reflects growing recognition that AD pathophysiology is multi-process, and that a multi-marker panel may better reflect heterogeneous trajectories than any single marker.

### 4.6 Implementation obstacles and existing problems

As BBMs move closer to clinical adoption, the dominant scientific challenge is shifting from discovery/association to implementation science. Firstly, Pre-analytical variation (choice of collection tube, time-to-processing, centrifugation protocols, storage conditions, freeze–thaw cycles) can introduce systematic bias that is large enough to move individuals across clinical decision thresholds. Work from the Standardization of Alzheimer’s Blood Biomarkers working group demonstrates that some analytes are sensitive to routine handling differences and highlights the need for standardized operating procedures, external quality control, and harmonized reporting of pre-analytical metadata (Verberk et al., 2022; Mantellatto Grigoli et al., 2024; Mitolo et al., 2024). Consequently, consensus work has emphasized that clinical implementation requires standardized protocols, multicenter validation, and prespecified cut points with prospective testing (Teunissen et al., 2022; Hansson et al., 2023). More recently, guideline-style recommendations have begun to formalize best practices for clinical use of BBMs, reflecting the maturation of the field toward regulated and routinized adoption (Palmqvist et al., 2025; Pahlke et al., 2025; Erickson et al., 2025; Mielke et al., 2024; Mielke and Fowler, 2024).

Second, the combination logic of multiple biomarkers needs to be explored. While p-tau and plasma Aβ anchor the “core pathology” dimension, our keyword clustering also points to sustained interest in neurodegeneration and astroglial activation markers. Plasma neurofilament light chain (NfL) is a sensitive marker of neuroaxonal injury and correlates with neurodegeneration but lacks disease specificity; it can be elevated in multiple neurological conditions (Mattsson et al., 2017; Teunissen et al., 2022). Plasma GFAP appears to capture astroglial activation and has shown stage-related patterns across the AD continuum, potentially improving early detection or risk stratification when integrated with core markers (Benedet et al., 2021; Teunissen et al., 2022). These observations support a generalizable principle: clinical utility often increases when biomarkers are combined to capture different biological dimensions (amyloid, tau, neurodegeneration, glial response) and to mitigate weaknesses of any single marker. Such multi-marker approaches also align with how A/T/N was designed—biological classification, not single-analyte diagnosis (Jack et al., 2018). Future bibliometric signals may therefore increasingly reflect not only “which biomarker wins,” but how marker combinations are optimized for specific clinical tasks such as triage, confirmation, staging, and monitoring.

Third, external validity and equity are central. There is increasing evidence that plasma biomarker distributions and interpretation may differ by race/ethnicity, comorbidity burden, and social determinants. Hajjar et al. reported race-associated differences in several plasma and CSF AD biomarkers, emphasizing that screening strategies relying on BBMs must account for group-level differences to avoid compounding underrepresentation in trials and inequity in care (Hajjar et al., 2022). Complementary work argues that medical comorbidities and ethnicity can impact plasma AD biomarkers, with direct implications for clinical trials and general clinical practice (O'Bryant et al., 2023). Beyond race/ethnicity, renal function, vascular risk, systemic inflammation, and other comorbidities may influence biomarker levels and thereby complicate “one-size-fits-all” cutoffs (Sarto et al., 2024). These findings suggest that the validation queue for Alzheimer’s disease blood markers must be diverse, with different gradient criteria rather than a single global threshold.

## 5 Limitations

This study has several limitations. First, although we used both WoSCC and Scopus, restricting data to these two databases and to English-language articles/reviews may still omit relevant studies indexed in other databases (e.g., PubMed, Embase) or regional databases, and may underrepresent non-English contributions. Second, bibliometric indicators are influenced by citation lag and field size; recent high-impact innovations may be undercounted in co-citation networks simply because they have not had sufficient time to accrue citations. Third, as a bibliometric analysis, this study describes publication patterns and citation networks but does not assess the methodological quality, clinical validity, or level of evidence of the included primary studies; the observed indicators should therefore be interpreted as reflections of scholarly attention rather than direct measures of clinical utility. Fourth, the exclusion of non-peer-reviewed literature, such as preprints and conference abstracts, was intended to maintain data consistency and quality, but this approach may introduce publication bias and could omit emerging findings that have not yet completed formal peer review. Fifth, because our retrieval was performed on 29 November 2025, the data for 2025 cover approximately 11 months rather than a full calendar year; the annual output for 2025 is therefore likely underestimated and should be interpreted with caution in temporal trend analysis. Therefore, interpretation should be integrated with systematic reviews and prospective clinical studies (Olsson et al., 2016; Teunissen et al., 2022; Hansson et al., 2023).

## 6 Conclusion

This bibliometric study mapped the global research landscape of AD BBMs from 2011 to 2025 (data retrieved up to 29 November 2025). The field has entered a rapid-growth, clinically translational stage, with a pronounced rise in output after 2018 and increasingly dense international collaborations. Plasma phosphorylated tau biomarkers, particularly p-tau217, constitute a major research frontier, accompanied by increasing emphasis on diagnostic performance, implementation recommendations, and multi-marker panels integrating Aβ, tau, neurodegeneration, and astroglial activation markers. These findings provide a data-driven “knowledge map” that may guide future research priorities and support evidence-based clinical translation.
