# Supplementary material for: Global research trends and hotspots in blood-based biomarkers for Alzheimer’s disease: a bibliometric analysis
Source: Front Aging Neurosci. 2026 Sep 29;18:1886781. doi: 10.3389/fnagi.2026.1886781 (PMC13659226; doi:10.3389/fnagi.2026.1886781)
Supplement: Supplementary file 1 [file Table_1.docx]

**Supplementary Table S1. Full search syntax for WoSCC and Scopus**

| **Database** | **Search Query** |
| --- | --- |
| WoSCC | TS=(("Alzheimer's disease" OR "Alzheimer disease")) AND TS=(("blood biomarker*" OR "plasma biomarker*" OR "serum biomarker*" OR "blood-based biomarker*" OR "plasma p-tau" OR "phosphorylated tau" OR "plasma amyloid" OR "plasma Aβ" OR "neurofilament light" OR "NfL" OR "GFAP" OR "glial fibrillary acidic protein")) |
| Scopus | TITLE-ABS-KEY("Alzheimer's disease" OR "Alzheimer disease") AND TITLE-ABS-KEY("blood biomarker*" OR "plasma biomarker*" OR "serum biomarker*" OR "blood-based biomarker*" OR "plasma p-tau" OR "phosphorylated tau" OR "plasma amyloid" OR "plasma Aβ" OR "neurofilament light" OR "NfL" OR "GFAP" OR "glial fibrillary acidic protein") |
| Time span | 1 January 2011 – 29 November 2025 |
| Language | English |
| Document types | Articles and reviews |

This expanded query served as the primary search strategy; the broad terms and analyte-specific terms were combined using OR, and no separate searches were performed.
